# *Livoneca redmanii* Leach, 1818 (Cymothoidae) a parasitic isopod infesting the gills of the European seabass, *Dicentrarchus labrax* (Linnaeus, 1758): morphological and molecular characterization study

**DOI:** 10.1186/s12917-022-03405-2

**Published:** 2022-08-31

**Authors:** Ebtsam Sayed Hassan Abdallah, Awatef Hamed Hamouda

**Affiliations:** 1 Department of Aquatic Animal Medicine and Management, Faculty of Veterinary Medicine, Assiut University, Assiut, 71526 Egypt; 2 Fish Health and Diseases Department, Faculty of Fish and Fisheries Technology, Aswan University, Aswan, 81528 Egypt

**Keywords:** *Livoneca redmanii*, Morphology, Phylogenetic analysis, *Dicentrarchus labrax*, Mediterranean Sea – Egypt

## Abstract

**Background:**

Prevalence, morphology, and molecular characteristics of isopodiosis in the European seabass, *Dicentrarchus labrax*, in Egypt were assessed using light and electron microscopy and polymerase chain reaction targeting the mitochondrial *COI* (cytochrome oxidase c) gene.

**Results:**

Adult parasites were found mainly in the branchial cavity between gill arches and to a lesser extent in the buccal cavity. They were morphologically identified as the Cymothoidae *Livoneca redmanii* Leach, 1818 (Crustacea: Isopoda). Obviously, a 23% prevalence rate of isopods was reported in *D. labrax* from Egyptian Mediterranean waters. Destructive and degenerative necrotic alterations with complete sloughing of gill lamellae of the infested fish were observed. DNA sequencing of the mitochondrial *COI* gene confirmed the identification of the parasite which was deposited in the GenBank under accession numbers MW600099, MZ208984, and MZ208985. Furthermore, the phylogenetic analysis demonstrated that parasites emerged from a monophyletic clade closely affiliated with *L. redmanii* and were clearly distinguished from other isopod genospecies.

**Conclusion:**

The present investigation addresses *L. redmanii* infestation in *D. labrax* in Egypt and affirmed morphological properties via the scanning electron microscopy (SEM) and molecular characteristics of this isopod species. The drastic effects of this parasite on the infected fish were proven both clinically and histopathologically.

**Supplementary Information:**

The online version contains supplementary material available at 10.1186/s12917-022-03405-2.

## Background

The European seabass, *Dicentrarchus labrax* industry is a major success story that began in the late 1980s, exploded exponentially in the 1990s, and continued to grow over the next two decades. Production in 2016, climbed from a few thousand to 191,000 t in less than 15 years [1]. In the Mediterranean region, Egypt is a large producer of *D. labrax* [1]. The production of seabass in the Mediterranean Sea is primarily based on hatchery-produced fingerlings. However, in some areas of Egypt, wild-caught fingerlings are employed in aquaculture [2]. As a result, if wild-caught fingerlings get infested with isopods, their introduction to aquaculture could threaten the lucrative seabass industry.

Parasitism has a negative effect on the health and productivity of fish [3]. Fish parasitic arthropods include species from the copepoda, branchiura, and isopoda. About 450 isopod species parasitize marine and freshwater fish [4]. Cymothoidae, Leach, 1818 (Crustacea: Isopoda) is a family of common and big ectoparasites found in brackish, freshwater, and marine environments [5]. Cymothoidae members attach themselves to buccal or branchial cavities, gills, and body surfaces causing significant tissue damage and mortality [6]. Cymothoidae is characterized by adaptive behavior for a parasitic lifestyle and shows great diversity in tropical and subtropical marine waters. In general, cymothoids are protandrous hermaphrodites that develop first into males and then females [7]. After turning female, parasites are unable to leave their host [8]. Adult cymothoid isopods are big aggressive-looking ectoparasitic crustaceans that are mostly host and site-specific [9]. Depending on the species and location of attachment, they produce varying degrees of tissue damage [10–12]. Branchial isopods have a significant negative impact on the heart and respiratory metabolism of fish [13].

*Livoneca redmanii* is a parasitic cymothoid isopod that parasitizes freshwater and marine fishes all over the world and is not native to the Mediterranean. Its previous range was limited to the eastern coast of the United States, from New York to Rio de Janeiro [13], and it had been found in eastern Australian marine fishes [14]. The parasite was recently discovered in a variety of hosts in the estuary waters of Lake Maracaibo, Venezuela [15], as well as in the Atlantic bumper, *Chloroscombrus chrysurus*, in Brazilian coastal waters [16]. In Egypt, *L. redmanii* was isolated from flathead gray mullet, *Mugil cephalus* [17], common sole, *Solea solea* in Lake Qarun [18], redbelly tilapia, *Tilapia zillii* [19], and cultured meager, *Argyrosomus regius*, in northern lakes [20]. Adult parasites are usually seen in male/female pairs in their host’s branchial cavities [21]. Free-swimming juveniles were surprisingly identified on mugiliid fry collected from the Mediterranean Sea and transported to Lake Qarun [22].

Standard morphological characteristics of body parts and appendages are used to identify cymothoid isopods into genera and species [23, 24]. Molecular techniques, such as nucleic acid amplification by polymerase chain reaction (PCR), nucleotide sequencing, and phylogenetic analysis, are also helpful. These latter methods allow for the detection of genetic variation within subspecies and strains [25, 26].

Because the knowledge about the prevalence of *L. redmanii* infestation, morphology, and molecular characteristics, as well as the impact of this isopod on the infested *D. labrax*, is still lacking, the ultimate goal of the current study was to obtain a precise identification of the current parasite that would aid in accurate diagnosis and subsequent effective disease control by 1) determining the seasonal prevalence of *L. redmanii* infestation, 2) examining the parasite’s morphological features using scanning electron microscopy (SEM), 3) assessing histological abnormalities in the infested *D. labrax*, and 4) conducting a phylogenetic analysis of the isolated *L. redmanii* from coastal waters of the Mediterranean Sea, Egypt.

## Methods

### Ethical statement

Fish were handled according to the standard protocol approved by Aswan University, Faculty of Fish and Fisheries technology, Department of Fish Diseases Ethics Committee for Animal Use, and Care (Number 5/2020, date 01.01.2020).

### Fish sampling, clinical signs, and postmortem investigations of the fish

**Fig. 1:**
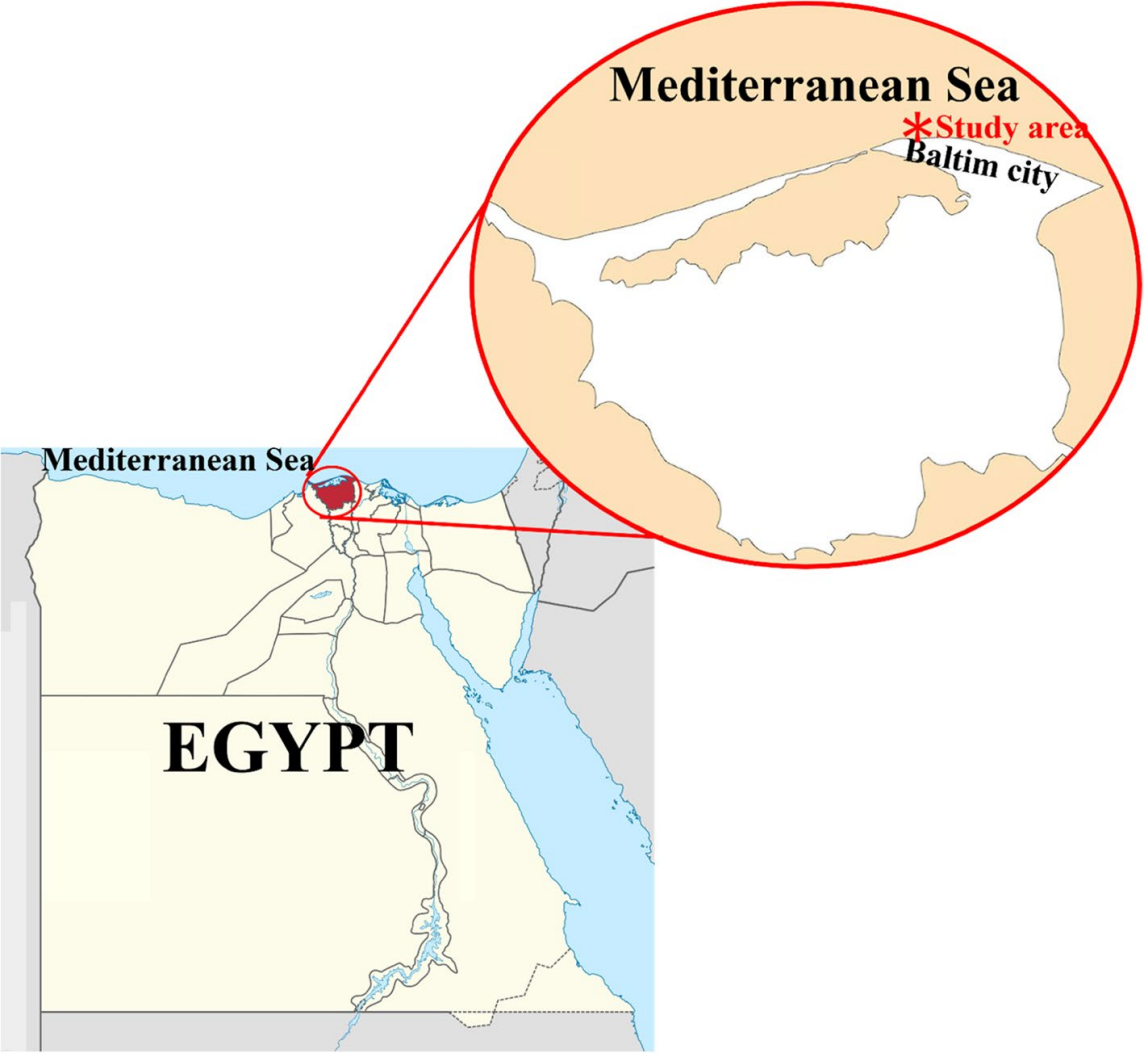
Study area

Two hundred wild, live *D. labrax* with a total length of 14.0 ± 0.5 cm were gathered from the Mediterranean Sea at Baltim District, Kafr Elsheikh Governorate, Egypt (Fig. 1), where fifty fish were sampled per season between March 2020 and February 2021. Fish were handled following standard protocol number (5/2020) approved by the Animal Use and Care Committee of the Faculty of Fish and Fisheries Technology, Aswan University, Egypt. They were directly evaluated for any visible clinical signs, and postmortem lesions, according to AE Eissa [27] and EJ Noga [28]. Fish were euthanized with clove oil [29] and examined as previously described by ES Hassan, MM Mahmoud, AM Metwally and DM Moktar [30].

### Parasitological examination

The body surface, fins, gills, opercula, and branchial and buccal cavities of each fish specimen were examined for parasitic isopods. Infested gill arches were dissected and investigated in a physiological saline solution. Recovered isopods were counted and cleaned numerous times in phosphate-buffered saline (PBS). The parasite length and width were measured to the nearest millimeter (mm), photographed, microscopically analyzed, and then preserved in 70% analytical grade ethanol. Parasites were morphologically identified according to RC Brusca [7], NE Mahmoud, MM Fahmy and MM Abuowarda [31]. Prevalence (number of infested fish/total number of examined fish × 100), the mean intensity (total number of parasites collected/total number of infested fish), and mean abundance (total number of parasites collected/ total number of examined fish) of the parasite through the whole study period (1 year) were calculated according to AO Bush, KD Lafferty, JM Lotz and AW Shostak [32].

### Scanning electron microscopy (SEM)

*Adult L. redmanii* isopods were removed and directly fixed for 24 h at 4 °C in 5% glutaraldehyde in 0.1 M phosphate buffer (pH 7.2). They were then rinsed in the buffer before being post-fixated in 1% osmium tetroxide for 2 h at 4 °C in the same buffer. Fixed specimens were dehydrated, and critical points dried using a sequence of escalating ethanol concentrations of 30, 50, 70, 90, and 100% for half an hour each. Subsequently, using the procedure described by ES Hassan, MM Mahmoud, AM Metwally and DM Moktar [30], they were sputter-coated with gold. The scanning electron microscope (JEOL 5400 LV) at the electron microscope unit (Assiut University, Egypt) was used to photograph the gold-coated parasites. Ten parasites were used for morphological investigation.

### Histopathological examination

Gills were prepared and examined as previously described (ES Hassan, MM Mahmoud, AM Metwally and DM Moktar [30]. Briefly, adult *L. redmanii* infested gill filaments were fixed in alcohol, formaldehyde, and acetic acid (85, 10, 5% respectively, AFA) solution. Thereafter, the parasite was preserved in 70% ethanol. Subsequently, specimens were dehydrated in a series of ethanol dilutions. The specimens were impregnated three times in melted paraffin wax before being embedded in melted paraffin. To analyze the pathology generated by the parasites, blocked tissues were sectioned (3–7 μm) using a Leica Microtome (Leica, Wetzlar, Germany), mounted using DPX, stained with hematoxylin and eosin, and viewed under a light microscope (Nikon Eclipse 80i, Japan), and photographed.

### Molecular characterization of the parasite

#### DNA extraction and PCR assay

DNA was extracted from a single parasite (*n* = 3) using the CTAB method as previously described (ESH Abdallah, MM Mahmoud and IR Abdel-Rahim [33]. A nanophotometer (Implen GmbH, Germany) was used to measure DNA concentration and purity at an optical density (OD) of 260 nm and a relative OD of 260/280 nm, respectively. Until used, DNA samples were stored at − 20 °C. PCR was conducted to amplify the 776-base pair (bp) segment of the mitochondrial cytochrome oxidase c subunit I (*COI*) using a thermal cycler (Veriti® model 9902; Applied Biosystems, USA) and the primer pair: LCO1490 (5′-GGTCAACAAATCATAAAGATATTGG-3′) and HCO2198 (5′-TAAACTTCAGGGTGACCAAAAAATCA-3′) (O Folmer, M Black, W Hoeh, R Lutz and R Vrijenhoek [34]. Amplified products were electrophoresed in 1.5% agarose gels in Tris-acetate EDTA (TAE) buffer, stained with 0.05 μg/ml ethidium bromide (Serva, Germany), and observed using a UV transilluminator (MultiDoc- It, UVP, UK). The size of the PCR products was determined using a 100 bp DNA ladder H3 RTU (GeneDireX). To purify PCR products from gels for sequencing, a Zymoclean Gel DNA Recovery Kit (Zymo Research, USA) was employed. The same amplification primers were used to sequence purified PCR products. SolGent Company Limited (Daejeon, South Korea) performed the DNA sequencing. The Basic Local Alignment Search Tool (BLAST) (http://www.ncbi.nlm.nih.gov/BLAST) was used to assess the results of a BLAST search conducted on the NCBI website.

#### Phylogenetic analysis

DNA Baser (version 5.15.0) was used to trim *CO1* gene sequences obtained in this investigation. The obtained *CO1* gene sequences were matched to closely comparable sequences in the GenBank to be relevant to Cymothoidae and then uploaded to the GenBank to provide accession numbers. The outgroup in this study was the *Phreatomerus latipes* haplotype (HM068160) a free-living isopod that lives in groundwater. The random stepwise addition of 1000 replicates was used in the maximum likelihood (ML) analysis [35]. The Kimura 2-parameter model and the Maximum Likelihood technique were used to infer the evolutionary history [36]. A total of 20 nucleotide sequences were used in this study. MUSCLE [37] was used to align all of the *CO1* gene sequences. Gaps and missing data were removed from all positions (complete deletion option). There was a total of 627 positions in the final dataset. The branch lengths were measured in the number of substitutions per site and the tree was drawn to scale (below the branches). MEGA X version 10.2.6 [38] was used to visualize the phylogenetic tree. Microsoft PowerPoint 365 was used to alter the tree which was then saved as a TIF file.

### Statistical analysis

The seasonal prevalence data were analyzed using a one-way analysis of variance (ANOVA; The Friedman test). Graph Pads Prism^®^ 8 Software was used to perform this analysis (version 8.4.3).

## Results

### Clinical signs

**Fig. 2:**
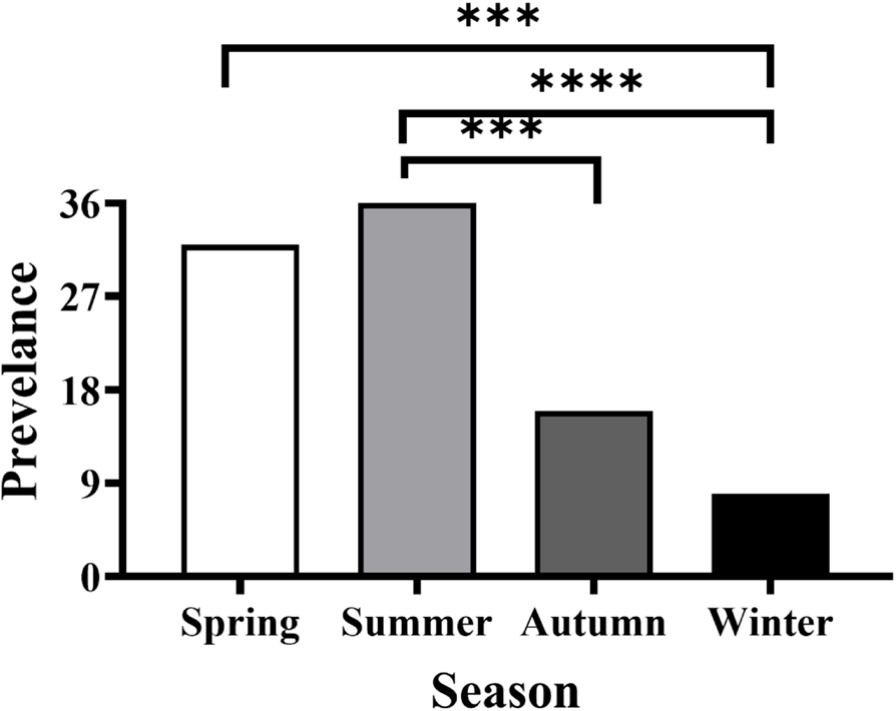
Seasonal prevalence of *Livoneca redmanii* in European seabass *Dicentrarchus labrax*

Adult *L. redmanii* was found in 46 of the 200 *D. labrax* tested (23% prevalence). The percentage of infested fish per season varied from 8 to 36%, with summer having the greatest significant prevalence values (36%) and winter having the lowest (8%). The Friedman test (*P* < 0.0001) confirmed that *L. redmanii* infestation had a distinct seasonal pattern, with significant variations between summer and both winter and fall (Fig. 2). Female fish had a greater infection rate (36.7%) than male fish (14.1%).

**Fig. 3:**
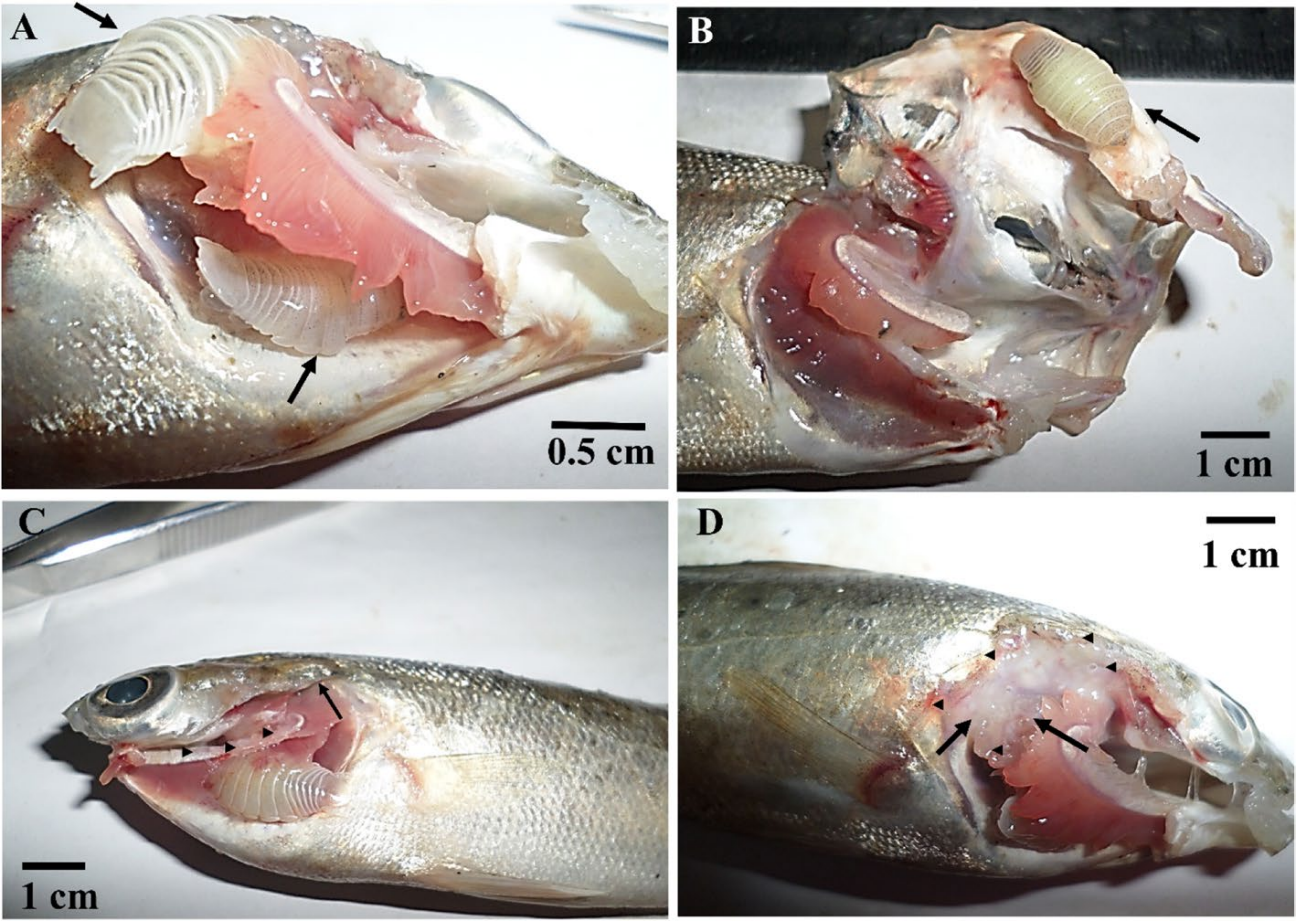
European seabass *Dicentrarchus labrax* naturally infested with adult *Livoneca redmanii* showing **A***L. redmanii* (arrows) attached to pale gill filaments and localized in the branchial cavity. **B***L. redmanii* (arrows) attached to the inner surface of the operculum. **C** Destruction and sloughing of the gill filaments (arrowheads). **D** Tissue destruction at the site of its attachment (arrowheads) with the destruction and paleness of the gill filaments (arrows)

The number of mature *L. redmanii* per fish ranged from one to three, with a mean intensity of 1.59 and a mean abundance of 0.365 (Table 1). Hypoxic fish with flaring opercula and agape mouths was the characteristic feature of *L. redmanii*-infested fish. Pale necrotic gills were also observed. Adult *L. redmanii* was found residing mainly in the branchial cavity, between gill arches (Fig. 3A and, C), or adhered to the inner surface of the operculum (Fig. 3B). Excessive mucus production was seen in the infested gill. After the manual removal of the parasites, pits were observed at the sites of the attachment (Fig. 3D).

**Table 1:** Prevalence, mean intensity, and mean abundance of adult *Livoneca redmanii* isolated from wild European seabass *Dicentrarchus labrax* caught from the Egyptian coastal area of the Mediterranean Sea

**Examined*D. labrax*number**	**200**
**Infested*D. labrax*number**	**46**
***L. redmanii*prevalence percent**	**23**
**Isolated*L. redmanii*number**	**73**
***L. redmanii*mean intensity**	**1.59**
***L. redmanii*intensity range**	**1–3**
***L. redmanii*mean abundance**	**0.365**

### Parasitological examination

**Fig. 4:**
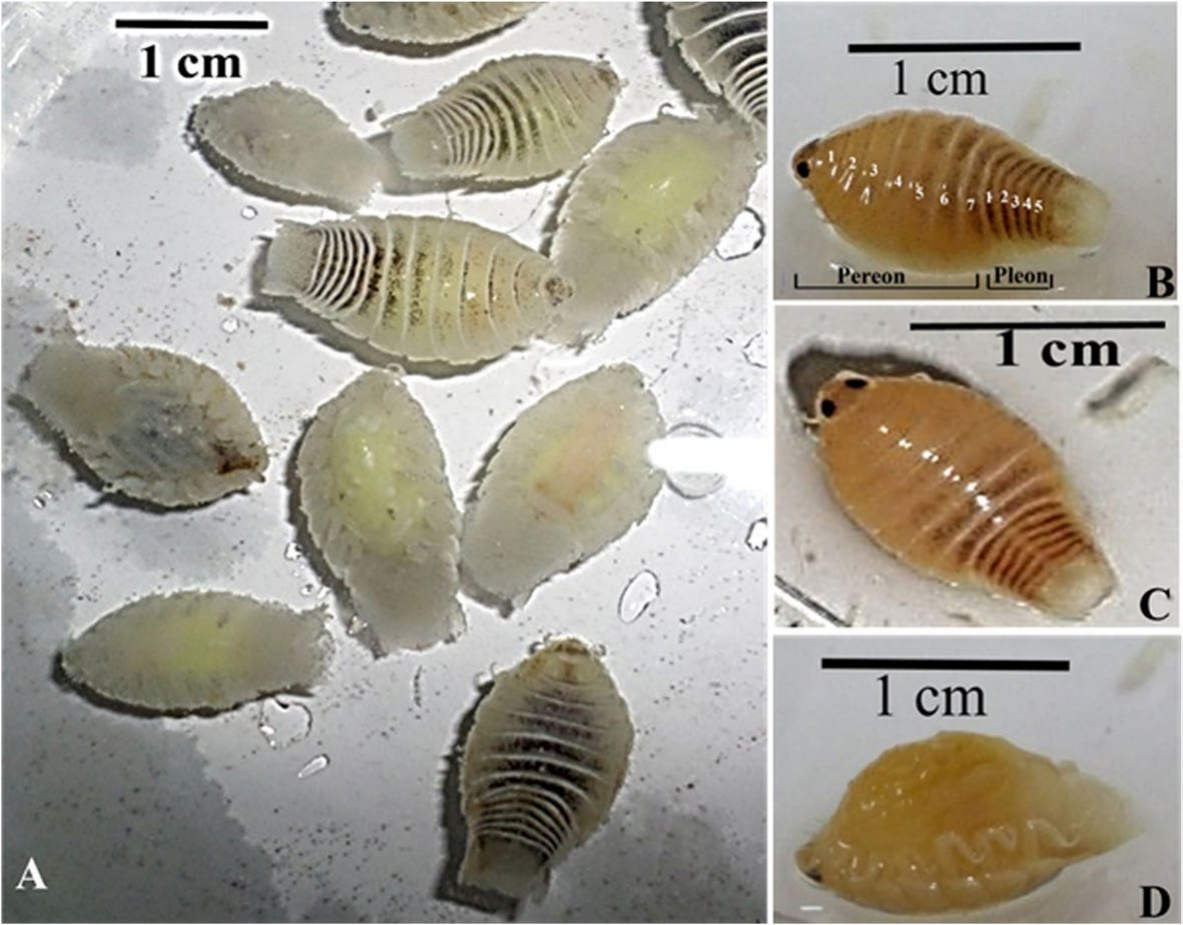
**A***Livoneca redmanii* collected from the branchial cavity of European seabass *Dicentrarchus labrax*; **B** and **C** dorsal aspects of adult *L. redmanii* showing pair of sensory eye, unsegmented cephalon, pereon (seven pereonites), and pleon (five pleonites); **D** ventral aspect of *L. redmanii* showing marsupium

**Fig. 5:**
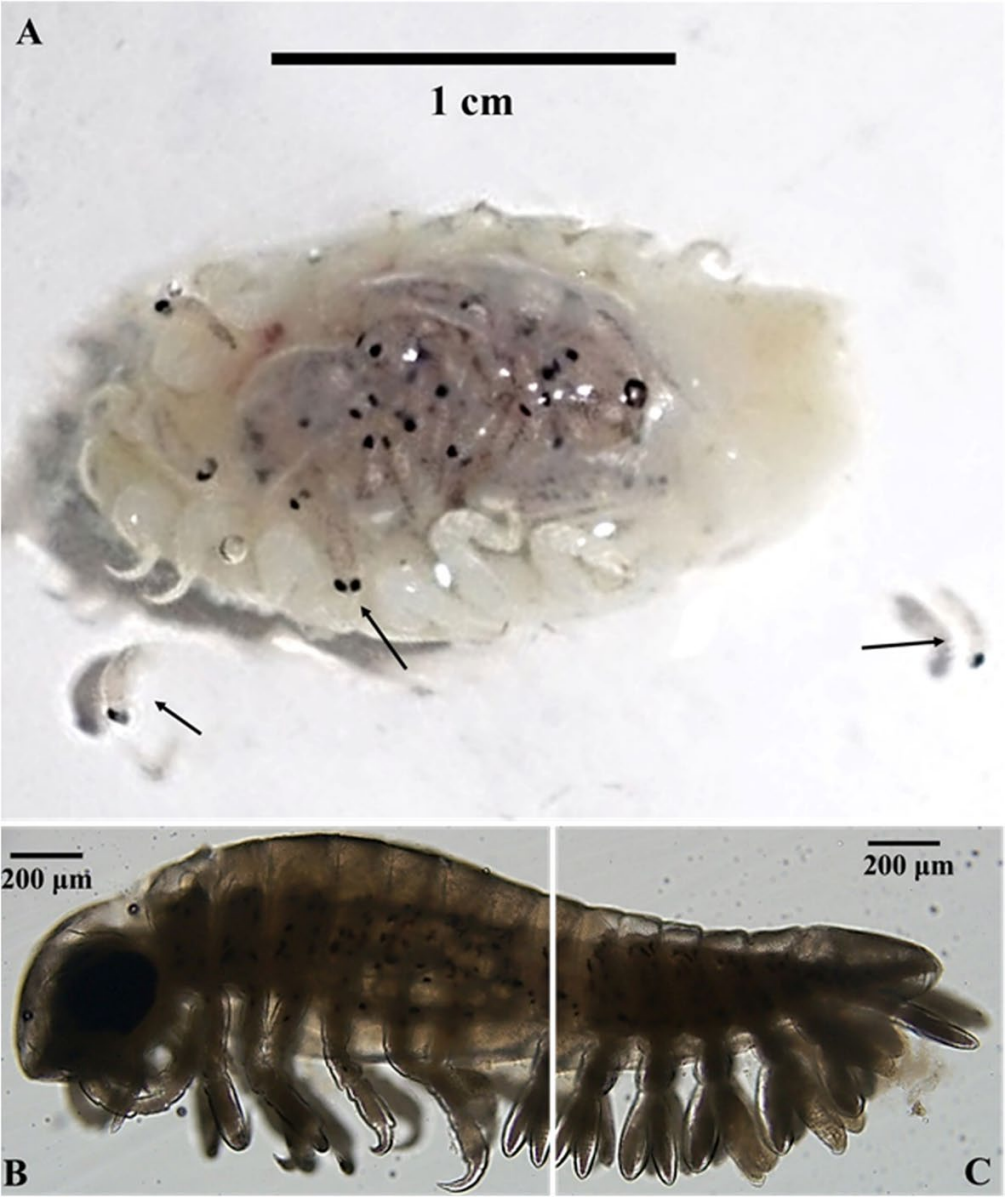
**A** Ventral aspect of the gravid female of *Livoneca redmanii* collected from the branchial cavity of European seabass *Dicentrarchus labrax* showing immature manca larvae inside the marsupium and released from the parasite (arrows); higher magnification of the whole body of the immature *L. redmanii* manca; **B** the anterior aspect; **C** posterior aspect of the immature *L. redmanii* manca

*L. redmanii* has a huge body size with average total lengths ± standard deviation of 19.3 ± 1.3, 15.8 ± 2.6 mm in female and male *L. redmanii* respectively. Adult female and male *L. redmanii* had an average width of 6.2 ± 0.2, and 5.4 ± 0.4 mm, respectively. Female parasites made up the majority of the separated parasites (78.1%), with male parasites accounting for only 21.9%. Adult males and females have identical macroscopic and microscopic characteristics in general. The body appears symmetrical and flattened dorso-ventrally. The head is non-segmented, and it has two distinct black eyes. The dorsal surface of fresh specimens was smooth creamy white, striated with grey lines with chromatophores (Fig. 4A). The body appears symmetrical and flattened dorso-ventrally. A non-segmented cephalon, pereon (seven pereonites, each with strong pigmentation near its posterior boundary), and pleon (five pleonites, each of which narrows in breadth as it moves towards the back) made up the body (Fig. 4B). A non-segmented head with two distinct well-developed black eyes was observed. The cephalon is concave and pointed with a folded anterior edge. The color of the samples preserved in ethanol or AFA altered to yellowish-brown with dark brown lines and chromatophores on the dorsum (Fig. 4B-D). The pouch (marsupium carrying young) on the ventral surface of gravid females was dark grey. Many black specks, the mancae’s sensory ocular organs, could be observed inside the pouch (Fig. 5A). Fifty mancae had an average length of 2.7 ± 0.3 mm. Mancae have six pairs of legs, big compound eyes, and pleopods with setae (Fig. 5B & C).

### Scanning electron microscopy of adult *L. redmanii*

**Fig. 6:**
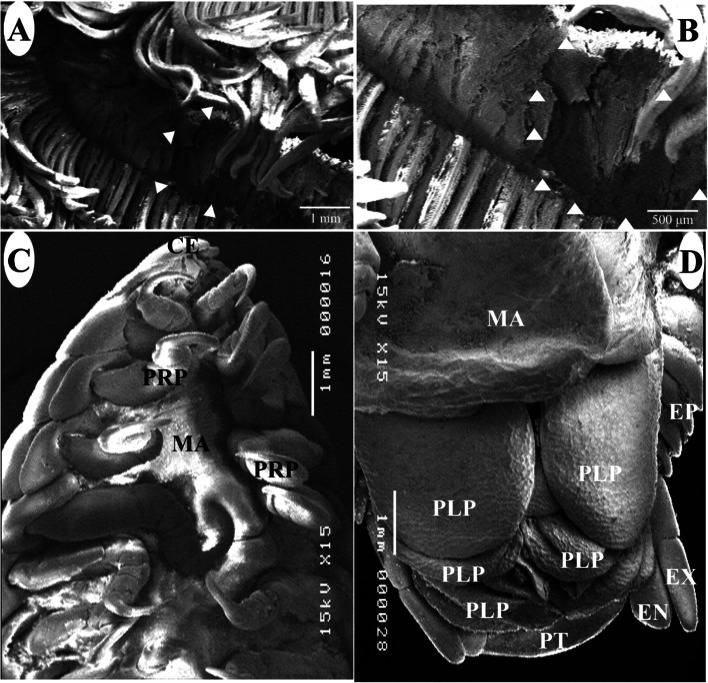
Scanning electron micrographs of *Livoneca redmanii* isolated from European seabass *Dicentrarchus labrax* showing: **A** Site of the attachment (arrowheads) of the adult parasite between 1st and 2nd-gill arches, with severe gill damage. **B** Higher magnification of the site of parasite attachment showing the destruction of gill lamellae. **C** Ventral view of the anterior parts of the female body region. **D** Ventral view of the posterior parts of the female body region. CE, cephalon; EP, epimera; PRP, pereopods; MA, marsupium; PLP, pleopods; PT, pleotelson; EN, endopodite of uropod; EX, exopodite of uropod

Between gill arches, the adult parasite was detected (Fig. 6A and B). There was considerable necrosis and tissue damage in the attachment area (Fig. 6A and B). The body consisted of a non-segmented cephalon, pereon, and pleon (Fig. 6C and D). The pereon which is made up of seven articulating pereonites, is the largest body region (Fig. 6C). On the ventral surface of each female is a marsupium or brood pouch, where the mancae are kept in this pouch (Fig. 6C and D). The pleon is situated behind the pereon. Pleopods, a pair of uropods, and a pleotelson are visible ventrally on the parasite (Fig. 6D). Exopodites of the uropod are taller than endopodites (Fig. 6D).

**Fig. 7:**
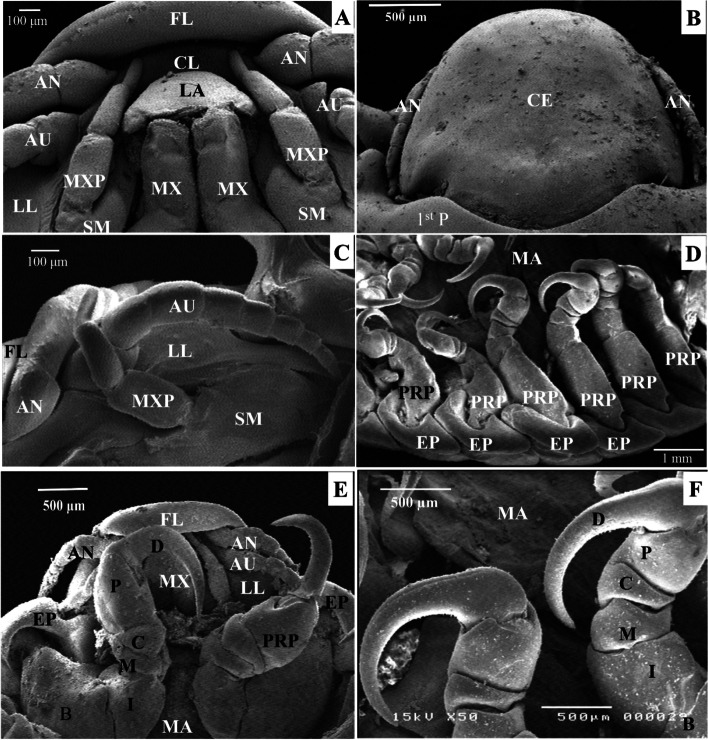
Scanning electron micrographs of a female *Livoneca redmanii* isolated from European seabass *Dicentrarchus labrax* showing **A** Cephalon showing mouthparts including maxilla, maxillipeds, and labrum, in addition to antenna and antennule; **B** dorsal view of the cephalic region showing cephalon, antenna, and first pereonite; **C** ventral view of dissected cephalon showing antenna, antennule, maxilliped, frontal lamina, and lateral lamina; **D** ventral view showing pereopods, epimera, and part of the marsupium; **E** ventral view of the anterior part of the parasite showing frontal lamina, antenna, antennule, maxilliped, first pair of the first pereopods, and part of the marsupium; **F** high magnification of the 4^th^ and 5th pereopods showing different parts of the pereopods including large basis, ischium, merus, carpus, propodus, and a large curved dactyl. AN, antenna; AU, antennule; CE, cephalon; CL, clypeus; FL, frontal lamina; LA, labrum; LL, lateral lamina; MX, maxilla; MXP, maxilliped; SM, stem of maxilliped; 1^st^ P, first pereonite; EP, epimera; B, basis; I, ischium; M, merus; C, carpus; P, propodus; D, dactyl; MA, marsupium

The cephalon is triangular and the first perionite does not cover it ( Fig. 7B). Ventrally, the cephalon is formed by a concave frontal lamina, a convex clypeus, two smooth lateral laminae, and medially located sucking mouthparts (Fig. 7A). The cephalon’s anterior edge is rounded (Fig. 7B). The sensing organs are carried by the cephalon, which includes two eyes, two antennae (eight segments), and two antennulae (eight segments, Fig. 7C). The antenna is longer than the antennulae and they both originate from the clypeus (Fig. 7C).

Single labrum, paired columnar maxillae, and two maxillipeds make up the mouthparts (Fig. 7A). The labrum is located on the medial side of the antenna and the antennulae. It has a convex upper surface, and a flat lower surface (Fig. 7A). The maxillae are columnar and are found medial to the maxillipeds and ventral to the labrum. Maxillipeds are formed from three segments and located lateral to the mouthparts (Fig. 7C). Each pereopod displays a large basis, ischium, merus, carpus, propodus, and a large, curved dactyl (Fig. 7D–F). The first three pairs of pereopods face backward, while the fourth, fifth, sixth, and seventh pairs face forward (Fig. 7D).

### Histopathological examination

**Fig. 8:**
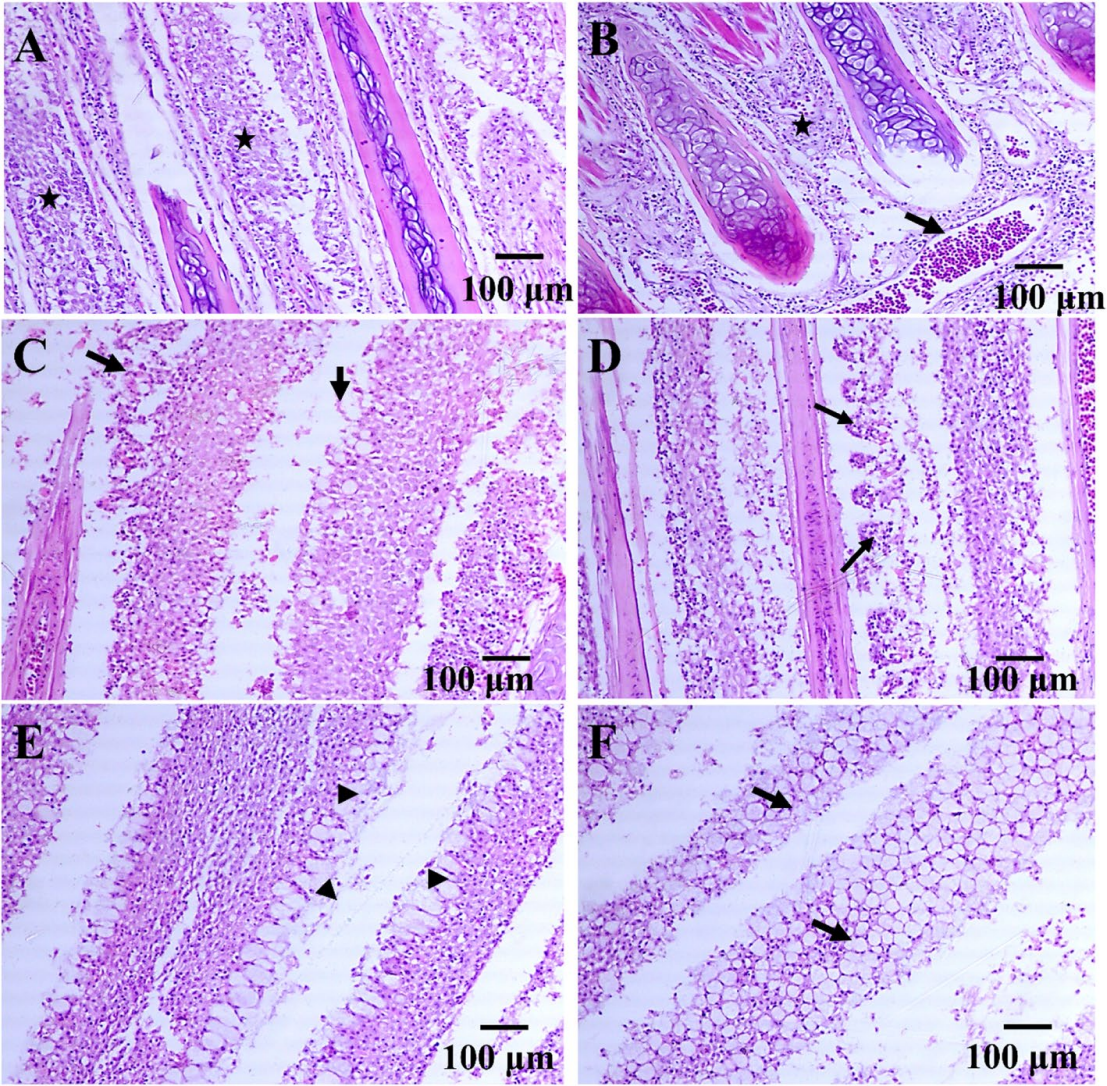
Histopathological alterations of the European seabass *Dicentrarchus labrax* gills induced by adult *Livoneca redmanii* showing: **A** marked inflammatory cell infiltration mostly granular eosinophilic cells, lymphocytes, and macrophages within the gill arch (stars), **B** inflammatory cellular infiltration (star) and vascular congestion (arrow), **C** degenerative necrotic changes and complete sloughing of the gill lamellae (arrows), **D** diffuse necrosis of gill lamellae (arrows), **E** mucus cell hyperplasia (arrowheads), **F** mucus cell hyperplasia in gill lamellae (arrows). X 100

Gill lamellae of infested fish exhibited marked inflammatory cell infiltration, particularly granular eosinophilic cells, lymphocytes, and macrophages within the gill arch ( Fig. 8A and B) as well as vascular congestion (Fig. 8B). Furthermore, in certain cases, degenerative necrotic alteration and complete sloughing of the primary gill lamellae were seen (Fig. 8C). Diffuse necrosis of gill lamellae (Fig. 8D) and mucus cell hyperplasia were seen in most of the other gill lamellae (Fig. 8E and F).

### Molecular characterization and phylogenetic analysis of *L. redmanii*

**Fig. 9:**
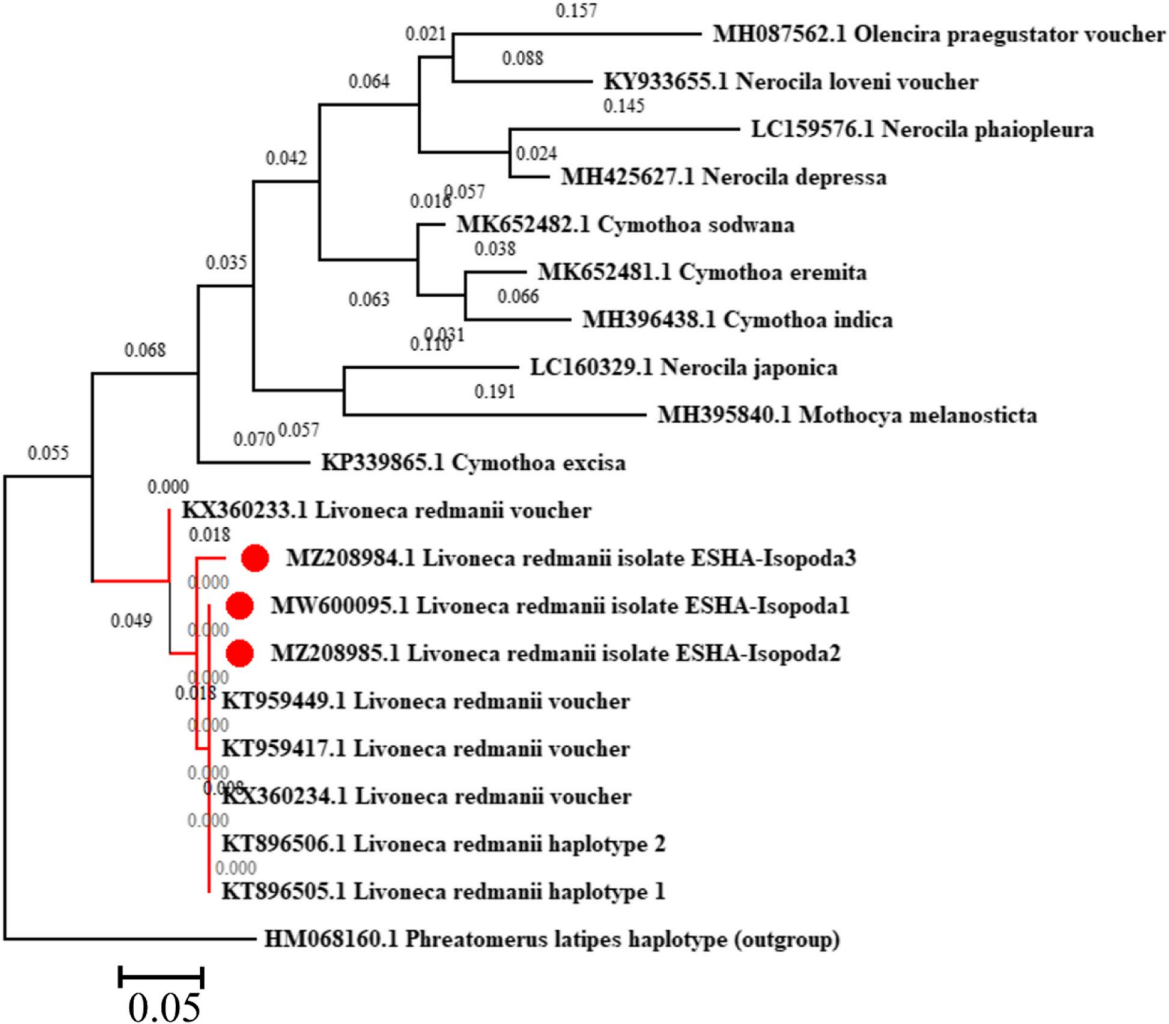
The Maximum likelihood method used to assess phylogenetic relationships among Cymothoidae and this study isolates of *Livoneca redmanii* (MW600099, MZ208984, and MZ208985) that were isolated from European seabass *Dicentrarchus labrax* based on the mitochondrial *COI* sequence using the MEGA-X program. The bar represents genetic distance due to sequence variation

An amplicon of approximately 776 bp (Supplementary data) was used to identify species using the mitochondrial *CO1* gene. Blast search using the obtained sequences resulted in 100% identity with the USA isolates (KT959417& KT959449), this study isolates, and the previously isolated Egyptian isolates (KT896505 & KT896506). Maximum likelihood analysis, using the Mega-X program, showed a close relationship between this study isolates and other strains isolated from either USA or even in the previous studies in Egypt that originated from a monophyletic clade that is closely affiliated to the genospecies *L. redmanii* (Fig. 9). On the other hand, *COIp*-distances between *L. redmanii* and the other Cymothoidae species included in this analysis were much higher (0.253–0.372; Table 2). In the present study, *COI* sequences were deposited in the GenBank on the NCBI under accession numbers MW600099, MZ208984, and MZ208985.

**Table 2:** Intraspecific and interspecific divergence distance among *COI* gene sequences of parasitic isopod species and the current study isolates (MW600099, MZ208984 & MZ208985)

	MW600095.1* Livoneca redmanii*	MZ208985.1* Livoneca redmanii*	MZ208984.1* Livoneca redmanii*	KT959449.1* Livoneca redmanii*	KT959417.1* Livoneca redmanii*	KX360234.1* Livoneca redmanii*	KX360233.1* Livoneca redmanii*	KT896506.1* Livoneca redmanii*	KT896505.1* Livoneca redmanii*	LC160329.1* Nerocila japonica*
**MW600095.1* Livoneca redmanii***										
**MZ208985.1* Livoneca redmanii***	**0.000**									
**MZ208984.1* Livoneca redmanii***	**0.015**	**0.005**								
**KT959449.1* Livoneca redmanii***	**0.000**	**0.000**	**0.005**							
**KT959417.1* Livoneca redmanii***	**0.000**	**0.000**	**0.005**	**0.000**						
**KX360234.1* Livoneca redmanii***	**0.003**	**0.001**	**0.005**	**0.001**	**0.001**					
**KX360233.1* Livoneca redmanii***	**0.009**	**0.006**	**0.012**	**0.006**	**0.006**	**0.004**				
**KT896506.1* Livoneca redmanii***	**0.000**	**0.000**	**0.005**	**0.002**	**0.002**	**0.002**	**0.009**			
**KT896505.1* Livoneca redmanii***	**0.000**	**0.000**	**0.005**	**0.000**	**0.000**	**0.000**	**0.007**	**0.002**		
**LC160329.1* Nerocila japonica***	**0.253**	**0.262**	**0.271**	**0.257**	**0.257**	**0.260**	**0.262**	**0.274**	**0.270**	
**MH087562.1* Olencira praegustator***	**0.284**	**0.287**	**0.300**	**0.280**	**0.280**	**0.282**	**0.282**	**0.286**	**0.286**	**0.263**
**MK652482.1* Cymothoa sodwana***	**0.279**	**0.287**	**0.283**	**0.278**	**0.278**	**0.275**	**0.277**	**0.278**	**0.278**	**0.249**
**LC159576.1* Nerocila phaiopleura***	**0.288**	**0.286**	**0.270**	**0.277**	**0.277**	**0.275**	**0.277**	**0.266**	**0.269**	**0.279**
**KP339865.1* Cymothoa excisa***	**0.242**	**0.279**	**0.285**	**0.279**	**0.279**	**0.276**	**0.276**	**0.283**	**0.283**	**0.242**
**MK652481.1* Cymothoa eremita***	**0.279**	**0.290**	**0.271**	**0.285**	**0.285**	**0.282**	**0.285**	**0.274**	**0.274**	**0.261**
**MH425627.1* Nerocila depressa***	**0.314**	**0.300**	**0.279**	**0.290**	**0.290**	**0.287**	**0.292**	**0.274**	**0.270**	**0.272**
**MH396438.1* Cymothoa indica***	**0.293**	**0.299**	**0.285**	**0.291**	**0.291**	**0.289**	**0.291**	**0.283**	**0.283**	**0.266**
**KY933655.1* Nerocila loveni***	**0.315**	**0.301**	**0.287**	**0.290**	**0.290**	**0.288**	**0.288**	**0.288**	**0.284**	**0.244**
**MH395840.1* Mothocya melanosticta***	**0.318**	**0.316**	**0.300**	**0.314**	**0.314**	**0.311**	**0.313**	**0.304**	**0.304**	**0.252**
**HM068160.1* Phreatomerus latipes***	**0.302**	**0.360**	**0.361**	**0.356**	**0.356**	**0.356**	**0.356**	**0.372**	**0.368**	**0.331**

	MH087562.1 *Olencira praegustator*	MK652482.1 *Cymothoa sodwana*	LC159576.1 *Nerocila phaiopleura*	KP339865.1 *Cymothoa excisa*	MK652481.1 *Cymothoa eremita*	MH425627.1 *Nerocila depressa*	MH396438.1 *Cymothoa indica*	KY933655.1 *Nerocila loveni*	MH395840.1 *Mothocya melanosticta*	HM068160.1 *Phreatomerus latipes*
**MW600095.1* Livoneca redmanii***										
**MZ208985.1* Livoneca redmanii***										
**MZ208984.1* Livoneca redmanii***										
**KT959449.1* Livoneca redmanii***										
**KT959417.1* Livoneca redmanii***										
**KX360234.1* Livoneca redmanii***										
**KX360233.1* Livoneca redmanii***										
**KT896506.1* Livoneca redmanii***										
**KT896505.1* Livoneca redmanii***										
**LC160329.1* Nerocila japonica***										
**MH087562.1* Olencira praegustator***										
**MK652482.1* Cymothoa sodwana***	**0.229**									
**LC159576.1* Nerocila phaiopleura***	**0.294**	**0.280**								
**KP339865.1* Cymothoa excisa***	**0.269**	**0.191**	**0.284**							
**MK652481.1* Cymothoa eremita***	**0.233**	**0.043**	**0.285**	**0.201**						
**MH425627.1* Nerocila depressa***	**0.295**	**0.275**	**0.130**	**0.267**	**0.295**					
**MH396438.1* Cymothoa indica***	**0.245**	**0.056**	**0.285**	**0.201**	**0.048**	**0.290**				
**KY933655.1* Nerocila loveni***	**0.241**	**0.236**	**0.233**	**0.248**	**0.258**	**0.234**	**0.251**			
**MH395840.1* Mothocya melanosticta***	**0.293**	**0.279**	**0.301**	**0.270**	**0.284**	**0.273**	**0.294**	**0.251**		
**HM068160.1* Phreatomerus latipes***	**0.352**	**0.331**	**0.331**	**0.329**	**0.343**	**0.317**	**0.326**	**0.306**	**0.340**	

## Discussion

European seabass is the most important fish species cultured in the Mediterranean areas [1]. With an annual production of 16,167 t in 2014, Egypt is a major seabass producer [39]. Despite Egypt’s long Mediterranean Sea shoreline (approximately 1000 km), there is little literature on *L. redmanii* in fish caught along the coast. As a result, the current study looked for *L. redmanii* infestation in *D. labrax*, an important marine fish in Egypt, as well as its negative impact on the fish. Furthermore, the isolated parasite has been identified both morphologically and molecularly to reach an accurate diagnosis of this parasite for future control strategies.

Seabass is subjected to a wide range of diseases, including isopodiosis. Isopods of the suborder Cymothoida consist of four superfamilies (Anthuroidea, Cryptoniscoidea, Cymothoidae, and Bopyroidea) that include more than 2700 defined species [40]. Cymothoidae members live in both freshwater and marine habitats, have a single-host holoxenic life cycle [41], and are very host and site-specific [10]. L Bunkley-Williams and EH Williams [10] described Cymothoids as permanent parasites that do not change or leave their hosts and thus parasitize their hosts chronically. *Ceratothoa oestroides*, *Nerocilla orbiguyi*, *Emetha audouini*, and *Anilocra physoides* are cymothoid parasites reported in seabass and cause severe clinical signs [1, 42–44].

In the current research, the sample size was chosen according to S Shvydka, V Sarabeev, VD Estruch and C Cadarso-Suárez [45] for accurate calculation of prevalence, mean intensity and mean abundance of a given fish parasite. The isolated isopod was identified as *L. redmanii* based on morphological traits previously described by NL Bruce [14], RC Brusca and GD Wilson [23]. Morphological characteristics were similar to those described for comparable parasites isolated from other Egyptian fish hosts, such as *A. regius* [20], *M. cephalus* [17], *S. solea* [18], mugiliid fry [22], and *T. zillii* [19].

Isopod abundance and dispersion are influenced by environmental factors such as water temperature, salinity, light intensity, predators, and food availability [46]. The total prevalence of *L. redmanii* in sampled *D. labrax* was 23% over the year, with the highest significant prevalence in the summer and the spring, and the lowest significant prevalence in the winter. High water temperatures in the summer and spring, as well as increased nitrite concentrations, are stressors that have been linked to high *L. redmanii* infestation rates in *T. zillii* [47]. Many isopod-related epizootics have been reported from the Turkish Mediterranean Coastal area [48], in Greece [44], among various Australian species [14], in South America in the estuarine waters of Lake Maracaibo, Venezuela [15], and among Brazilian species [10, 16, 49]. *Nerocila orbignyi* was discovered infesting 69% of randomly screened *D. labrax* [42]. In Egypt, *L. redmanii* was found in 46.7% of *M. cephalus* [17], 20.30% of *S. solea* in Lake Qarun [18], 10.6% of mugiliid fry collected from the Mediterranean Sea and transported to Lake Qarun [22], and an overall infestation rate of 19% among the studied fish species (*T. zillii*, *Solea* sp., and *Mugil capito*) with *M. capito* having the highest infestation rate (36%) [47]. In addition, prevalence rates of *L. redmanii* in cultured *A. regius* in Egyptian northern lakes were 77.1, 77.9, and 78.9% in Al-Madiyyah, Sidi Krier, and El Matareya, respectively [20]. *T. zillii* infested with *L. redmanii* had a 66% prevalence [19]. Similarly, isopodiosis has been reported in a variety of fish species. *Norileca orbignyi* was isolated from *T. zillii* in Lake Qarun, with a prevalence of 25% [50], as well as 33.5, 41.1, and 80.9%, from *T. zillii*, *S. solea*, and *M. capito*, respectively [51]. *M. melanosticta*, another isopod, attacked the red sea fish, *Nemipterus randalli*, in Egypt, with a prevalence of 40.96% [52]. The difference in prevalence could be due to the host or sampling area being different and or the water quality and temperature [47]. Isopodiosis can vary in frequency and severity [53], wherein the current investigation, *L. redmanii* parasitic load ranged from one to three per fish, with a mean intensity of 1.59 and a mean abundance of 0.365. Only one *L. redmanii* mean intensity per host *Chloroscombrus chrysurus* with a relative abundance of 0.05 parasites per fish captured [16] was recorded. The majority of the parasites recovered in this study were female. This is in agreement with S Ravichandran, G Rameshkumar and T Balasubramanian [41] who reported that the non-swimming, permanently attached adult females are the phases most commonly found cymothoids.

In this study, an adult *L. redmanii* with a massive body size up to 21 mm was isolated, causing substantial damage to the infested fish. Cymothids range in length from 0.5 to 440 mm [41] and have large leg hooks that cause wounds and limit fish growth [10]. The big, hefty body of *L. redmanii* causes gill filament pressure atrophy, crater formation in the branchial cavity at attachment sites, displacement of gill arches, and flared opercula. Such damage might be caused by biting and sucking mouthparts [54]. Furthermore, being a member of Cymothoids, *L. redmanii* has piercing-sucking mouthparts that allow it to feed on whole blood, seeping plasma from the wound, and macerated gill tissues [10]. The pale gills of infested *D. labrax* in the current investigation were consistent with this feeding behavior as feeding can result in anemia and impaired circulation due to vascular obstruction caused by huge isopods’ physical pressure [10].

The attachment sites of Cymothoidae are different depending on the fish species. It may attack the skin, fins, gills, and mouth, with some species, occasionally found trapped in fish musculature [55, 56]. We isolated adult *L. redmanii* from the branchial cavity, between gill arches, and attached it to the inner surface of the operculum. Residence in the buccal cavity was less frequent. Affected fish displayed respiratory manifestations such as hypoxia with open mouths, which were attributed to infection-related damage which could influence respiration efficiency and fish survival. Lesions detected in the infested fish’s gills confirmed the clinical findings. These findings are consistent with those of G Rameshkumar and S Ravichandran [54] who found *L. redmanii* attached to the inner surface of the opercula resulted in minor abrasion and hemorrhagic patches, along with gill and branchial damage. *L. redmanii* was lodged in the gill chamber or outside the operculum, in the mouth area, and at the ventrum of the head of the cultured meagre *A. regius* according to A Fadel, M Bessat and M Abdel-Aziz [20] and the gills eventually turned a pale tint, with extensive ulceration at the attachment site. In addition, maxilliped action during feeding has been linked to decreased respiratory competence [57–60]. Thus, pressure atrophy, feeding activity, and method of *L. redmanii* attachment to gills may all play a role in the clinical signs and histopathological alterations recorded in the infested fish [61, 62]. Also, *Emetha audouini*, a cymothoid isopod, has been discovered parasitizing cage-cultured *D. labrax* in large numbers in the buccal and branchial cavities of cage-cultivated *D. labrax* [44].

The infested *D. labrax* with *L. redmanii* produced an extraordinary amount of mucus in the current study. This could be the result of parasite feeding activity which has been associated with persistent irritation culminating in mucus cell hyperplasia, atrophy, and gill filaments and rakers loss [53, 63]. Furthermore, parasites cause significant damage and pressure necrosis to the occupied organs, both directly and indirectly, by biting and sucking as well as deformity and growth retardation. The latter causes fish to be unfit for food consumption [54]. The growth of *D. labrax* with two or more *C. oestroides* in the buccal cavity is substantially slower than that of fish with one or no parasites [42]. *L. redmanii* has been found detected in pairs in the gill chambers of cero, *Scomberomorus regalis*, and serra Spanish mackerel, *S. brasiliensis* (Osteichthyes: Scombridae), causing significant gill damage and possibly death, resulting in significant loss of these valuable species [10, 49]. *Nerocila orbignyi* was collected from the branchial cavity, buccal cavity, and lateral body surface of *D. labrax*, suggesting these areas have been proposed as predilection sites on this host [42].

In the current study, one to three mature parasites were detected in the infested *D. labrax* with *L. redmanii*. Cymothoids are protandrous hermaphrodites, that grow into males first and then females later [10]. Female parasites often prevent males from sexual transforming resulting in stable female-male pairings [10]. A marsupium or brood pouch is present in the ventral region of all gravid females. The young are held in the pouch until they mature into mancae, and there is no larval stage [10]. We isolated gravid females with free-living offspring who continued to molt into mancae. Mancae have six pairs of legs and pleopods with setae that allow them to swim swiftly and adhere to their definitive host after being released [64], providing a constant source of disseminating infection to both wild and cage-cultured seabass along the Egyptian Mediterranean coast. Juveniles may adhere to a paratenic host for a short time before a definitive host is found. After attaching to its definitive host, a juvenile loses its swimming setae and begins to develop into a male initially. Later transforming into female [10]. Other isopod genera, such as gill inhabiting forms *Anilocra*, *Nerocila*, *Mothocya*, certain *Livoneca* spp., tissue dwellers *Ourozeuktes* spp., and “tongue biters” *Ceratothoa* spp., exhibit this frequent transformation behavior [60, 65].

The morphological studies of the family Cymothoidae still face a challenge for differentiating among species [56] and cymothoids may be difficult to identify at the species level for non-taxonomists, therefore, molecular markers are of great importance in taxonomic terms [20, 48]. Molecular identification of *L. redmanii* using the mitochondrial *COI* gene sequence yielded a 776 bp amplicon, that confirmed its morphological identification [20, 48]. Phylogenetic analysis of the *L. redmanii* sequences obtained in the present study revealed a close relationship with the isolates from the United States, with 100% identity and 100% query sequence coverage supporting the hypothesis that this isopod is an alien species native to the western Atlantic and Caribbean [14]. All of the present isolates, USA isolates, as well as the previously identified Egyptian isolates originated from a single clade that can be distinguished from other Cymothoidae members. In addition, the *CO1 gene p*-distance among all *L. redmanii* isolates is significantly far from other Cymothoidae isopods.

## Conclusions

This study provides a definitive identification of *L. redmanii* using integrated morphological and molecular techniques which is essential for implementing preventative and control measures against this parasite. *L. redmanii* as an obligatory gill crustacean parasite was isolated from the branchial cavity causing severe damage with an obvious histopathological alteration in the gills of wild *D. labrax* caught off the Egyptian coast of the Mediterranean Sea. *L. redmanii*, a permanent nonswimming large parasite, adversely affects both the health and quality of *D. labrax*. The parasite’s negative effect on infested fish should be investigated further at the gene level. Infested *D. labrax* serves as a reservoir and a persistent source of infection, for both wild and farmed seabass along the Egyptian Mediterranean coast, posing a severe threat to Mediterranean mariculture. As a result, caution should be exercised while using wild-caught seabass fingerlings in aquaculture. Other research is needed to understand the interspecific link between these parasites and additional molecular identification utilizing more genetic markers and/or regions as the isolated parasites had 100% molecular similarities to the Egyptian and USA isolates.

## Supplementary Information

**Additional file 1.** 

## Data Availability

Data and materials are available upon reasonable request from the corresponding author. The datasets generated during the current study are available in the GenBank under accession numbers MW600099, MZ208984, and MZ208985.
