# Supplementary material for: Livoneca redmanii Leach, 1818 (Cymothoidae) a parasitic isopod infesting the gills of the European seabass, Dicentrarchus labrax (Linnaeus, 1758): morphological and molecular characterization study
Source: BMC Vet Res. 2022 Aug 31;18:330. doi: 10.1186/s12917-022-03405-2 (PMC9434925; doi:10.1186/s12917-022-03405-2)
Supplement: Supplementary file 1 — Additional file 1. [file 12917_2022_3405_MOESM1_ESM.docx]

**Supplementary data**

Electrophoretic analysis of PCR-amplified mitochondrial *CO1* gene of *Livoneca redmanii* infesting European seabass *Dicentrarchus labrax.* Lane L: Molecular weight marker (100-bp DNA ladder), Lanes 1, 2, and 3: positive samples (776 bp), Lane 4: no template control
